# Starch to protein ratio and food moisture content influence water balance and urine supersaturation in cats

**DOI:** 10.1371/journal.pone.0315949

**Published:** 2024-12-18

**Authors:** Maria Eduarda Gonçalves Tozato, Stephanie de Souza Theodoro, Leticia Warde Luis, Lucas Bassi Scarpim, Pablo da Cunha Costa, Ana Paula Judice Maria, Gener Tadeu Pereira, Aulus Cavalieri Carciofi

**Affiliations:** Departament of Veterinary Medicine and Surgery, School of Agricultural and Veterinary Sciences, São Paulo State University (UNESP), Jaboticabal, São Paulo, Brazil; University of Agriculture Faisalabad, PAKISTAN

## Abstract

Two starch to protein ratios (high starch [HS], 25% starch and 36% protein; high protein [HP], 15% starch and 53% protein on DM basis) and two moisture contents (5%, dry kibbles; 80%, wet food) were compared in a 2 x 2 factorial arrangement totaling 4 diets. Each diet was evaluated in 9 cats, with 8 d of total collection of urine and feces. Results were subjected to an analysis of variance of the effects of starch to protein ratio, moisture content and their interactions (P<0.05). Urine density was lower and volume was higher in cats fed wet foods (P<0.01). Calcium (Ca) urine concentration was higher for dry and HP diets (P<0.05). The oxalate urine concentration was 60% higher for cats fed both HS formulations (dry and wet; P<0.05). The relative supersaturation (RSS) of urine for calcium oxalate was higher for dry foods and HS formulations (P<0.01), and for struvite, it was lower for both wet foods, and among the dry diets, it was lower for the HS than for the HP formulation (P<0.01). Foods with a high protein-to-starch ratio reduced urine oxalate and RSS for calcium oxalate in wet and dry diets, and wet foods reduced RSS for calcium oxalate and struvite.

## Introduction

Urolithiasis is a complex biomineralization process that starts with the formation of microcrystals and ends with mature urolith development in the urinary tract [1,2]. Many controversies exist about the etiology and the role of diet in the development or prevention of urolith formation [3–5], but adequate nutrition for urolith-forming cats is considered essential to avoid recurrences and invasive procedures to remove stones [6].

Dietary intake and its metabolism will influence the renal excretion of calculogenic substances, which together with water balance and the consequent urine volume will determine the concentration of substances in the urine that may promote or prevent mineralization [7,8]. Urine pH is another important factor strictly controlled by diet macroelement composition [9,10] that determines the force of aggregation and crystal biomineralization [11]. However, the use of urine pH alone is not adequate to predict urolith formation, which is better accessed by the calculation of the relative supersaturation of the urine (RSS) [11,12].

Dry kibble diets, which are very popular among cat owners, result in lower water intake via food, as they typically have less than 10% moisture. Although some controversial results have been published [13–15], most recent studies suggest that cats do not compensate for this low moisture in food and exhibit low total water intake and consequently reduced urine volume and increased urine density in comparison with animals consuming wet foods or dry foods supplemented with more than 70% water [15,16]. However, although some data are available [8,12,16], the impact of this dilution of urine promoted by wet food consumption has not yet been fully explored with regard to urine RSS for calcium oxalate and struvite.

Dry kibble diets are also relatively high in starch content in comparison with prey, the diet of ancestral felines [17]. Starch is necessary for extrusion process and kibble formation [18]. If properly gelatinized, starch becomes digestible, and is a source of energy for cats [19,20]. However, high starch intake may result in less urine production, as protein intake is known to increase renal water excretion and diuresis in cats [21,22]. High protein intake induces elevated production of urea, which increases tonicity in the glomerular filtrate, water retention and urine volume. As urea increases urine osmolality, although a larger amount of urine is produced, its osmolality and density remain high [23]. Additionally, increased calcium (Ca) renal excretion in cats fed high protein formulations has been reported [24] and thus the impact of the increased urine volume induced by protein intake on the urine RSS of cats is not completely known.

However, increased starch intake results in higher metabolic availability of glucose, a precursor of oxalate, through the glyoxylate pathway [25]. Therefore, high-starch diets have been implicated in increasing endogenous oxalate synthesis in feline hepatocytes [26]. This hypothesis is supported by a study that demonstrated lower urine volume, higher oxalate urine concentration and higher urine RSS for calcium oxalate in cats fed high starch diets in comparison with those fed high protein dry diets [5]. This effect of starch intake, however, is still incompletely understood, as another study failed to demonstrate higher endogenous oxalate synthesis in cats fed a high starch formulation [27].

Given that the intake of wet foods induces higher total water intake and urine volume, the hypothesis of the present study is that regardless of the starch to protein ratio of the formulation, the intake of wet foods will reduce urine RSS for calcium oxalate and struvite in comparison with the intake of dry foods. However, in dry foods, lower starch intake, obtained by feeding high protein formulations, will result in lower endogenous synthesis and renal excretion of oxalate and higher urine volume. Therefore, although high protein intake may increase renal Ca excretion, it will lower urine RSS for calcium oxalate. To test these hypotheses, the present study evaluated the water balance, urine characteristics, and renal excretion of oxalate, calcium, and citrate and measured the urine RSS for calcium oxalate and struvite in cats fed dry or wet foods with two starch-to-protein ratios.

## Materials and methods

### Ethical aspects and experimental design

The experiment was performed at the Research Laboratory in Nutrition and Nutritional Diseases of Dogs and Cats “Prof. Dr. Flávio Prada”, College of Agrarian and Veterinarian Sciences, São Paulo State University (UNESP), Jaboticabal, Brazil. The experimental procedures were were approved by the Ethics Committee in the Use of Animals of the São Paulo State University (Unesp), School of Agricultural and Veterinarian Sciences, Jaboticabal, São Paulo, Brazil (protocol number 3501/20). The cats live in a collective cattery with two internal rooms and an outdoor playground. There are responsible employees for animal care, and a veterinary technical in charge for the colony. Cats have daily contact with staff and students.

Thirty-six mixed-breed neutered cats, 4.1±1.2 years old, with a mean body condition score (BCS) of 5.3±0.6 on a scale of 9 points [28] were used. Before the study, each cat was physically examined by a veterinarian, and hematological and serum biochemical profiles (albumin, alkaline phosphatase, alanine aminotransferase, urea and creatinine) and complete urine analysis were conducted, indicating good health.

The experiment was conducted using a completely randomized block design, with cats divided into 3 blocks of 12 animals and 3 cats eating one of the 4 experimental foods in each block, totaling 9 cats per diet. The blocking factor was period, due to the impossibility of testing all cats at the same time. In each block, cats received the diets for 18 d, 10 d for adaptation followed by 8 d of total collection of feces and urine. During adaptation, the cats were restricted to individual cages (0.65x0.80x0.65 m) for 16 h (from 16:00 to 8:00) and placed in a cattery for 8 h to exercise and for socialization (from 8:00 to 16:00). The cages were stainless steel with an apparatus for the separate collection of feces and urine. All cats interacted with people at least three times per day: to be housed and fed, to be released into the cattery and to be brushed for 15 min. During the feces and urine collection periods, cats were restricted to their cages. The cages were washed daily, rinsed with distilled water and dried. Distilled water was provided ad libitum.

To control food consumption, diets were only available when the animals were in their cages. Cats were fed twice daily, one meal offered at the morning (7:00 hs) and the second in the afternoon (16:00 hs). The food intake was individually calculated. Food metabolizable energy was estimated from their chemical composition, and cats were initially fed 75 kcal/kg^0.^67/day [29]. The offered amount and leftovers were weighed at each meal, and the intake was recorded. Food amount was controlled to cats’ body weight remain constant along the study. To this, animals were weighed weekly in the morning before the meal, and the amount offered was adjusted if the body weight of the cat varied more than 5% in comparison with the initial value.

### Experimental diets

The treatments were organized in a 2 x 2 factorial arrangement, composed of 2 starch to protein ratios and 2 food moisture contents, totaling 4 experimental diets. Diets were formulated to meet the nutrient requirements of adult cats [29]. Two treatments were formulated to present high starch (approximately 25% on a dry matter basis) and moderate crude protein (approximately 36%) contents, resulting in an elevated starch-to-protein ratio. The other two treatments were formulated to present high protein (approximately 52%) and low starch (less than 15%), resulting in a low starch to protein ratio. Each proposed starch to protein ratio was further divided into two raw material sources and processing types: extruded dry food or canned high moisture pouches (wet food). The high starch dry food and the two wet diets were manufactured at ADIMAX Company (Salto de Pirapora, São Paulo, Brazil). The high protein dry food was manufactured at the Extrusion Laboratory of UNESP, Jaboticabal, Brazil.

Due to the strong influence of urine pH on RSS [5], before formulation, the ingredients were purchased and analyzed for dry matter, crude protein, starch, Na, K, Ca, Mg, Cl, P and S. These analyses were performed according to procedures recommended by the AOAC [30]. Based on the obtained results, foods were formulated to meet the protein and starch objectives and to present a similar food base excess (BE; approximately -20 mEq/kg of DM) targeting a urine pH close to 6.2 [10]. Food BE was calculated based on the analyzed results of each raw material according to the following equation: BE (mEq/kg of DM) = (49.9 x Ca) + (82.3 x Mg) + (43.5 x Na) + (25.6 x K)—(64.6 x P)—(62.4 x S)—(28.2 x Cl).

After production, the diets were analyzed for dry matter, ash, crude protein, acid-hydrolyzed fat, starch, crude fiber, and minerals following recommended procedures of the AOAC [30]. The amino acid content was analyzed by high-performance liquid chromatography [31,32] and tryptophan was analyzed according to the method of Lucas & Sotelo [33]. The analyzed chemical composition of the experimental diets as well as their ingredients are presented in Table 1.

**Table 1:** Analyzed chemical composition of experimental diets for cats with different starch to protein ratios and moisture contents. Values on dry matter basis.

Item	Dry foods		Wet Foods	
	High starch^1^	High protein^2^	High starch^3^	High protein^4^
Moisture (%)	4.78	4.74	81.61	81.61
	**%, DM basis**			
Starch	28.75	15.33	25.32	6.69
Crude Protein	36.89	53.67	36.91	53.31
Essential amino acid				
Arginine	2.14	2.58	1.88	2.76
Histidine	0.81	1.08	0.95	1.25
Isoleucine	1.51	2.21	1.55	2.30
Leucine	2.53	3.79	2.96	4.43
Lysine	2.03	2.16	2.34	3.36
Methionine	0.76	0.93	0.68	0.93
Methionine + Cysteine	1.24	1.84	1.41	2.05
Phenylalanine	1.52	2.50	1.80	2.74
Phenylalanine + Tyrosine	2.54	3.88	2.84	4.23
Threonine	1.26	1.71	1.54	2.29
Leucine	2.53	3.79	2.96	4.43
Serine	1.13	2.05	1.37	2.17
Valine	1.74	2.49	1.98	2.97
Tryptophan	0.34	0.52	0.45	0.61
Taurine	0.48	0.40	0.45	0.42
Dispensable amino acids				
Glycine	2.75	3.16	2.09	2.86
Hydroxyproline	0.67	0.60	0.21	0.26
Aspartic acid	2.76	3.21	2.98	4.39
Glutamic acid	5.38	13.00	5.30	8.43
Proline	2.39	4.89	2.15	3.39
Tyrosine	1.02	1.38	1,78	1.49
Cystine	0.48	0.91	0.73	1.12
Alanine	2.05	2.35	1.85	2.68
Acid-hydrolyzed fat	17.01	17.47	24.06	23.92
Crude fiber	2.38	2.95	1.81	1.04
Ash	9.08	6.01	9.11	7.97
Calcium	1.57	0.68	1.15	1.16
Phosphorus	1.22	0.58	0.98	1.17
Sodium	0.91	0.79	1.13	1.41
Potassium	0.75	0.77	0.59	0.64
Magnesium	0.09	0.06	0.07	0.09
Chlorine	1.71	1.60	1.22	1.51
Metabolizable Energy (kcal/g of DM) ^5^	4.27	4.53	4.51	4.47

^1^ Ingredients: Broken rice, Poultry by-product meal, Poultry fat, Wheat gluten, Wheat bran, Swine-isolated protein, Sugarcane fiber, Beet pulp, Palatant enhancer, Common salt, Potassium chloride, Vitamin-mineral Premix, Choline chloride, Calcium carbonate, Taurine, Salmon oil, Mold Inhibitor, Antioxidant, DL-Methionine, Urine acidifier (Equilibrium 3D, SPF do Brasil, Descalvado. Composition: Common salt, DL-methionine, ammonium chloride, ammonium sulfate, sodium tripolyphosphate, citric acid, vegetable oil).

^2^ Ingredients: Wheat gluten, Poultry by-product meal, Broken rice, Poultry fat, Swine-isolated protein, Sugarcane fiber, Beet pulp, Palatant enhancer, Common salt, Potassium chloride, Wheat bran, Vitamin-mineral Premix, Choline chloride, Calcium carbonate, Taurine, Salmon oil, Mold Inhibitor, Antioxidant, DL-Methionine, Urine acidifier (Equilibrium 3D, SPF do Brasil, Descalvado. Composition: Common salt, DL-methionine, ammonium chloride, ammonium sulfate, sodium tripolyphosphate, citric acid, vegetable oil), Phosphoric acid.

^3^ Ingredients: Water, Chicken meat, Swine kidney, Cassava starch, Swine liver, Swine Plasma, Wheat gluten, Sugarcane fiber, Beet pulp, Xanthan gum, Common salt, Potassium chloride, Vitamin-mineral Premix, Choline chloride, Calcium carbonate, Taurine, Salmon oil, Mold Inhibitor, Antioxidant, DL-Methionine, Liquid Palatant, Urine acidifier (Equilibrium 3D, SPF do Brasil, Descalvado. Composition: Common salt, DL-methionine, ammonium chloride, ammonium sulfate, sodium tripolyphosphate, citric acid, vegetable oil).

^4^ Ingredients: Water, Chicken meat, Swine kidney, Swine liver, Swine Plasma, Wheat gluten, Sugarcane fiber, Beet pulp, Xanthan gum, Common salt, Potassium chloride, Vitamin-mineral Premix, Choline chloride, Calcium carbonate, Taurine, Salmon oil, Mold Inhibitor, Antioxidant, DL-Methionine, Liquid Palatant, Urine acidifier (Equilibrium 3D, SPF do Brasil, Descalvado. Composition: Common salt, DL-methionine, ammonium chloride, ammonium sulfate, sodium tripolyphosphate, citric acid, vegetable oil).

^5^ Food metabolizable energy was determined *in vivo*, with 9 cats per food, with the method of total collection of feces and urine [29].

### Digestibility and water balance

The feces were quantitatively collected at least twice a day for 8 d, weighed and frozen (-20°C). At the end of collection, the feces were thawed and homogenized to produce a single sample per cat. The fecal samples and wet diets were dried in a forced-air oven at 55°C for 72 h. The predried feces and diets were then ground in a cutting mill fitted with a 1 mm screen sieve prior to analysis. Moisture, crude protein, acid hydrolyzed fat, and starch were determined according to AOAC [30] proposed methodologies. The crude energy content was analyzed using a bomb calorimeter (IKA calorimeter, C200; IKA-Werke GmbH & Co. KG, Staufen, Germany).

Digestibility was calculated according to the recommendations of FEDIAF [29]. During collection, fecal samples were scored using the following system: 0 = watery liquid (pourable); 1 = soft, unformed; 2 = soft, malformed stool that assumes the shape of container; 3 = soft, formed, and moist, but retains shape; 4 = well-formed and consistent stool that does not adhere to the floor; and 5 = small, hard, dry pellets [34].

Water balance was determined by quantifying water intake at the drinker, water intake via food, metabolic water, water excretion via feces, and water excretion via urine. Insensible water losses were estimated by the difference in the total water intake (from drink, food, and metabolic water) and the losses via urine and feces [23]. Offered water and water remaining were weighed, allowing the calculation of daily intake. To quantify the water loss by evaporation at the drinker, one drinking bowl was kept in the same room as the cats, but without animal access, and the evaporative water was estimated and discounted in the calculation of the daily intake. The metabolic water was calculated by multiplying the amount of digestible protein consumed by 0.396, digestible starch by 0.566 and digestible fat by 1.071 [35].

### Urinary parameters and relative supersaturation for calcium oxalate and struvite

Urine was quantitatively collected in plastic bottles placed under the metabolic cages for 8 consecutive days. In the first 5 d, 100 mg of thymol (Synth, LABSYNTH, Diadema–SP, Brazil) was added to the bottles as a preservative. The urine produced was homogenized, and its volume was measured. The density (T2-Ne Clinical; ATAGO CO., LTD.; Fujita Yorii-cho Osato gun, Saitama, Japan) and pH (Digimed DM20; Digicrom Analítica, São Paulo, Brazil) of the urine were determined. Urine samples were further divided into two aliquots for storage: an acidified urine sample, prepared by the addition of 1 mL of 6 N HCl for each 50 mL of urine and stored at -20°C; and a nonacidified urine, which was frozen at -20°C. In addition, a 1 mL aliquot of each urine sample (acidified and nonacidified) was stored at -80°C for oxalate and citrate analyses. The concentrations of calcium, phosphorus, magnesium, urea and oxalate were determined in the acidified urine sample. Sodium, potassium, chloride, sulfur, citrate and uric acid were analyzed in the nonacidified sample.

Commercial kits (Labtest Diagnóstica S. A, Lagoa Santa, Brazil) were used to determine calcium, phosphorus, magnesium, uric acid, chloride and urea with a spectrophotometer (Labquest, Labtest Diagnóstica S. A, Lagoa Santa, Brazil). Sodium and potassium levels were determined using ion-selective electrodes (Roche Diagnostics, 9180, Indianapolis, USA). Sulfur was measured by the turbidimetric method [30] in a spectrophotometer after sample digestion with a nitric-perchloric solution (Model B442, Micronal, São Paulo, SP, Brazil). Citrate and oxalate were analyzed by enzymatic and colorimetric determination (Citric Acid and Oxalic Acid, LTA, Bussero, Milan, Italy) in a semiautomatic spectrophotometer (Labquest, Labtest Diagnóstica S.A., Lagoa Santa, MG, Brazil). On the last 3 d of total urine collection, 1 mL of 1 N H_2_SO_4_ solution was added to the collection bottles, and urine was collected at least 3 times a day and frozen (-20°C) for ammonia analysis. The urine sample was diluted in 1:4 formic acid solution, and ammonia was determined by the Kjeldahl method [30] with titration using a 2% boric acid solution.

Based on the results, the urine RSS for calcium oxalate was calculated using the software EQUIL-93 (Department of Biochemistry and Molecular Biology, University of Florida, USA), and the RSS for calcium oxalate and struvite was calculated using the software SUPERSAT (RSS calculator for pets, Mars Incorporated, updated version of the program developed by Robertson [36].

### Statistical analysis

For statistical analysis, the error normality hypothesis was evaluated using the Cramer‒von Mises test, and the homoscedasticity of variance was determined with the Levene test. The experimental unit was one cat. The data were analyzed in a randomized block design with 3 blocks of 12 cats, 3 cats per diet in each block and 9 cats (repetitions) per diet in total. Treatments were organized in a factorial arrangement with 2 starch to protein ratios and 2 food moisture contents, totaling 4 treatments. Data were subjected to an analysis of variance, and the model sums of squares were separated into the effects of starch to protein ratio, moisture content and their interactions (P<0.05). When differences were detected by F test and the interaction between starch to protein ratio *versus* moisture content was significant, the means of the four diets were compared with the Tukey test (P<0.05). The MIXED procedure of SAS software was used (version 9, SAS Inst. Inc., Cary, NC, USA).

## Results

The diets presented an adequate chemical composition (Table 1) with crude protein close to the target level. The starch content was similar for the high starch wet and dry formulations; however, the wet food high protein formulation presented a lower starch value than planned. Amino acid composition varied according to the raw material source and crude protein content. Mineral composition differed between diets because authors chose to study practical formulations and not to design semi-purified foods in which the minerals composition could be better controlled. Especially the lower ash, Ca, P, Na, and Mg amount of the high protein dry food is explained by the inclusion of wheat gluten as the main protein source, a purified protein source with low ash content. The higher Ca, P and Mg content of the high starch dry food is explained by the high inclusion of poultry by-product meal, a protein source that includes some bone material and more elevated ash, Ca, P and Mg. To try equalizing these macroelements, purified sources such as calcium carbonate and magnesium oxide could be added to the high protein dry food, however this cationic raw materials would increase urine pH of the cats [10]. This macroelements would also have different bioavailability than the ones of the bone material, potentially creating other interferences and due this authors opted to not use them. All nutrient levels were in accordance with the recommendations for cat maintenance [29].

Cats fed the dry foods were heavier (P<0.05), with no difference between the starch to protein ratio (P>0.05), as shown in Table 2. Therefore, the values of water and nutrient intake are presented per kg of body weight per day. If this was calculated as per cat per day a statistical difference related to cat size, and not to dietary treatment would appear interfering on results interpretation. Cats appropriately accepted the four diets, and all the animals remained healthy, with no episodes of gastrointestinal disturbance or altered behavior, and the body weight of the cats remained constant throughout the study (P>0.05). The mean energy intake along the study was 75.6 ± 7.0 kcal/kg^0.75^/day, without differences among diets (P>0.05). The dry matter intake was lower for cats fed wet foods (P<0.01), but dry matter intake did not differ between high starch and high protein formulations. The starch and protein intake differed between groups, based on the chemical composition of the treatments (P<0.05). An interaction between starch to protein ratio and moisture content was observed for starch intake (P<0.05), that was higher for high starch dry, then high starch wet, then high protein dry, and lower for high protein wet food (P<0.05).

**Table 2:** Body weight, and dry matter, crude protein and starch intake of cats fed experimental diets with different starch to protein ratios and moisture contents.

Item	Starch to protein ratio		Mean	P value	
	High starch	High protein		S:P^2^	Moisture
Body weight (kg)					
Wet food	3.59±0.24	3.60±0.24	3.59±0.20		
Dry food	4.24±0.25	4.47±0.24	4.36±0.20		
Mean	3.92±0.20	4.07±0.20		0.563	< .001
Dry Matter intake (g/kgBW/day)					
Wet food	10.0±0.42	9.6±0.42	9.80±0.30		
Dry food	11.5±0.42	11.1±0.42	11.30±0.30		
Mean	10.8±0.30	10.3±0.30		0.320	0.001
Crude Protein intake (g/kgBW/day)					
Wet food	3.70±0.12	5.24±0.13	4.47±0.09		
Dry food	4.26±0.12	5.76±0.13	5.01±0.09		
Mean	3.98±0.08	5.50±0.09		<0.001	0.001
Starch intake (g/kgBW/day)*					
Wet food	2.46±0.09^A^^b^	0.56±0.09^B^^b^	1.52±0.07		
Dry food	3.20±0.09^A^^a^	1.62±0.09^B^^a^	2.41±0.08		
Mean	2.83±0.08	1.09±0.08		< .001	< .001

^1^ SEM—standard error of the mean (n = 9 cats per food).

^2^ S:P—effect of the starch to protein ratio of the diet formulation.

* Starch to protein ratio * moisture content interaction (P<0.05).

^A,B^ Means in a row without a uppercase letter in common are different (P<0.05).

^a,b^ Means in a column without a lowercase letter in common are different (P<0.05). Comparison valid only within each parameter.

In general, the total tract apparent digestibility of nutrients was adequate (Table 3). No effect of the starch to protein ratio was observed for the nutrient digestibility coefficients or fecal characteristics (P>0.05). A statistical interaction between starch to protein ratio and moisture content was observed (P<0.05), with higher protein digestibility for the high starch wet food and lower crude fat and crude energy digestibility for the high protein wet food (P<0.05). The fecal output (as-is) did not differ significantly between cats fed the different diets (P>0.05). However, on a DM basis, fecal production was lower for high-starch wet food compared to high-protein wet food and higher for high-starch dry food compared to high-protein dry food (P<0.05). Regarding processing types, fecal DM excretion was greater for high-starch dry food than for high-starch wet food (P<0.05).

**Table 3:** Coefficients of total tract apparent digestibility of nutrients and feces characteristics of cats fed experimental diets with different starch to protein ratios and moisture contents.

Item	Starch to protein ratio		Mean	P value	
	High starch	High protein		S:P^2^	Moisture
Total tract apparent digestibility (%)					
Dry Matter					
Wet food	87.51±0.80	82.63±0.80	85.07±0.56		
Dry food	83.20±0.80	87.30±0.75	85.25±0.55		
Mean	85.35±0.56	84.98±0.55		0.626	0.772
Crude Protein*					
Wet food	93.70±0.51^A^^a^	91.13±0.54^B^^a^	92.40±0.40		
Dry food	90.06±0.54^A^^b^	90.55±0.54^A^^a^	90.31±0.39		
Mean	91.86±0.38	90.84±0.40		0.060	<0.001
Acid-hydrolyzed fat*					
Wet food	91.25±1.10^A^^a^	87.40±1.17^A^^b^	89.33±0.80		
Dry food	92.29±1.10^A^^a^	93.84±1.03^A^^a^	93.06±0.75		
Mean	91.77±0.77	90.62±0.78		0.302	0.002
Crude Energy*					
Wet food	87.82±1.00^A^^a^	83.48±1.00^B^^a^	85.65±0.81		
Dry food	86.75±1.00^A^^a^	88.48±1.02^A^^a^	87.62±0.81		
Mean	87.29±0.81	85.98±0.81		0.135	0.027
Feces characteristics					
g/kgBW/day, As-is					
Wet food	3.37±0.31	4.13±0.31	3.75±0.22		
Dry food	4.45±0.31	3.18±0.31	3.82±0.22		
Mean	3.91±0.22	3.65±0.22		0.423	0.838
g/kgBW/day, DM basis*					
Wet food	1.19^B^^b^±0.11	1.52^A^^a^±0.11	1.36±0.09		
Dry food	1.93^A^^a^±0.10	1.33^B^^a^±0.10	1.63±0.09		
Mean	1.56±0.09	1.43±0.09		0.116	0.002
Score					
Wet food	3.87±0.08	3.63±0.07	3.74±0.06		
Dry food	3.85±0.07	3.92±0.07	3.89±0.05		
Mean	3.86±0.05	3.77±0.05		0.247	0.060

^1^ SEM—standard error of the mean (n = 9 cats per food).

^2^ S:P—effect of the starch to protein ratio of the diet.

* Starch to protein ratio * moisture content interaction (P<0.05).

^A,B^ Means in a row without a uppercase letter in common are different (P<0.05).

^a,b^ Means in a column without a lowercase letter in common are different (P<0.05). Comparison valid only within each parameter.

Urine pH was similar among treatments (P>0.05), according to the formulation objective, to avoid a possible interference with RSS results (Table 4). An interaction between starch to protein ratio and food type was observed for urine density, water intake from food, metabolic water, total water intake, urine excretion (P<0.05). Urine density was higher for cats fed dry foods in comparison with cats fed wet foods, (P<0.01) and among cats fed dry foods, urine density was higher for high protein than for high starch diet (P<0.05). Water intake from food and total water intake were higher for cats fed wet foods (P<0.01), and within this diet type, it was higher for high protein than for the high starch formulation (P<0.05). In contrast, cats fed dry foods drank more water than cats fed wet foods (P<0.01), and there was no effect of the starch to protein ratio. The urine excretion of cats fed wet foods was more than double that of cats fed dry foods (P<0.01), and within the wet foods, urine production was higher for cats fed the high protein formulation than for cats fed the high starch formulation (P<0.05). The fecal water excretion corresponded to a small proportion of total water loss, and did not differ among treatments (P>0.05).

**Table 4:** Urine characteristics and water balance of cats fed experimental diets with different starch to protein ratios and moisture contents.

Item	Starch to protein ratio		Mean	P value	
	High starch	High protein		S:P^2^	Moisture
Urine characteristics					
Density (g/l)*					
Wet food	1.020 ±0.002^A^^b^	1.019±0.001^A^^b^	1.020±0.001		
Dry food	1.046 ±0.001^B^^a^	1.056±0.001^A^^a^	1.051±0.001		
Mean	1.033±0.001	1.038±0.001		0.001	< .001
pH					
Wet food	6.10±0.05	6.07±0.05	6.08±0.04		
Dry food	6.12±0.05	6.25±0.05	6.19±0.04		
Mean	6.11±0.04	6.16±0.04		0.358	0.055
Water balance (ml/kgBW/day)					
Water from food*					
Wet food	43.44±1.04^B^^a^	54.70±1.04^A^^a^	49.07±0.07		
Dry food	0.58±0.98^A^^b^	0.53±1.04^A^^b^	0.56±0.07	< .001	< .001
Mean	22.01±0.07	27.61±0.07			
Drinking water					
Wet food	0.17±0.93	0.19±0.93	0.10±0.81		
Dry food	20.90±0.96	20.69±0.93	20.80±0.83		
Mean	10.37±0.82	10.44±0.82		0.913	< .001
Metabolic water*					
Wet food	5.10±0.15^A^^a^	4.30±0.15^B^^a^	4.70±0.11		
Dry food	5.00±0.15^A^^a^	4.90±0.16^A^^a^	4.95±0.11		
Mean	5.05±0.11	4.60±0.11		0.012	0.111
Total water intake*					
Wet food	49.43±1.54^B^^b^	59.10±1.45^A^^a^	54.26±1.06		
Dry food	28.44±1.45^A^^a^	27.55±1.45^A^^b^	28.00±1.03		
Mean	38.94±1.06	43.32±1.03		0.010	< .001
Urine water excretion*					
Wet food	28.15±1.33^B^^a^	39.90±1.22^A^^a^	34.03±1.02		
Dry food	15.02±1.22^A^^b^	16.06±1.22^A^^b^	15.54±1.00		
Mean	21.59±1.02	27.98±1.00		< .001	< .001
Fecal water excretion					
Wet food	2.07±0.22	2.58±0.22	2.32±0.16		
Dry food	2.30±0.22	1.75±0.22	2.03±0.16		
Mean	2.19±0.16	2.17±0.16		0.935	0.190
Insensible losses					
Wet food	16.46±1.06	16.18±0.94	16.32±0.71		
Dry food	10.69±0.94	9.32±0.94	10.00±0.66		
Mean	13.57±0.71	12.75±0.66		0.403	< .001

^1^ SEM—standard error of the mean (n = 9 cats per food).

^2^ S:P—effect of the starch to protein ratio of the diet.

* Starch to protein ratio * moisture content interaction (P<0.05).

^A,B^ Means in a row without a uppercase letter in common are different (P<0.05).

^a,b^ Means in a column without a lowercase letter in common are different (P<0.05). Comparison valid only within each parameter.

An interaction between the starch to protein ratio and food type was observed for Ca intake (P<0.05). Calcium intake was similar between cats when wet foods were compared, but it was significantly higher (P<0.05) in cats fed the high starch dry diet than in those given the high protein dry formulation. Regarding formulation type, Ca intake was higher with the high starch dry formulation and higher with the high protein wet food (P<0.05; Table 5). However, Ca urine concentration and renal excretion were higher for cats fed the high protein formulations, even though Ca intake was lower for cats fed these diets (P<0.05). For sodium (Na) intake an interaction among starch to protein ratio and food type was observed (P<0.05), with higher intake for cats fed high protein wet food, followed by high starch wet and dry foods, and lower for cats fed the high protein dry food (P<0.05). An interaction was observed for Na urine concentration, which was higher for cats fed dry foods, regardless of starch to protein ratio. Within the dry foods, urinary Na concentration was higher in cats fed the high starch formulation compared to the high protein formulation (P<0.05). Na renal excretion was higher for cats fed wet foods and the high protein formulations (P<0.05). The urine concentration of uric acid was higher for cats fed dry foods (P<0.01) and was similar for the high starch and high protein formulations. However, uric acid renal excretion was higher for cats fed the high protein formulations and wet foods (P<0.01).

**Table 5:** Intake, urine concentration, and renal excretion of calcium, sodium, potassium, chloride, magnesium, phosphorus and metabolites of cats fed experimental diets with different starch to protein ratios and moisture contents.

Item	Starch to protein ratio		Mean	P value	
	High starch	High protein		S:P^2^	Moisture
Ca intake (mg/kgBW/d)*					
Wet food	113.9±4.69^A^^b^	107.3±4.69^A^^a^	110.6±3.94		
Dry food	176.6±4.86^A^^a^	63.5±4.69^B^^b^	120.1±4.00		
Mean	145.3±4.00	85.4±4.03		< .001	0.014
Ca urine concentration (mg/dL)					
Wet food	1.43±0.37	1.49±0.37	1.46±0.26		
Dry food	4.03±0.35	5.64±0.35	4.83±0.25		
Mean	2.73±0.25	3.56±0.25		0.029	< .001
Ca renal excretion (mg/kgBW/d)					
Wet food	0.46±0.07	0.59±0.07	0.52±0.05		
Dry food	0.65±0.07	0.82±0.08	0.73±0.05		
Mean	0.55±0.05	0.70±0.05		0.037	0.005
Na intake (mg/kgBW /d)*					
Wet food	113.96±4.17^B^^a^	135.83±3.94^A^^a^	124.89±2.87		
Dry food	105.6±3.9^A^^a^	87.9±3.94^B^^b^	96.7±2.78		
Mean	109.91±2.87	111.88±2.78		0.626	< .001
Na urine concentration (mg/dL)*					
Wet food	299.5±14.75^A^^b^	286.0±14.75^A^^b^	292.7±10.43		
Dry food	543.9±18.07^A^^a^	439.8±14.75^B^^a^	491.9±10.66		
Mean	421.7±11.66	362.9±10.43		0.001	< .001
Na renal excretion (mg/kgBW/d)					
Wet food	93.2±7.65	115.5±7.65	104.4±5.41		
Dry food	66.2±7.65	75.8±7.65	71.0±5.41		
Mean	79.7±5.41	95.6±5.41		0.045	0.001
Uric Acid urine concentration (mg/dL)					
Wet food	4.63±0.61	5.00±0.61	4.81±0.43		
Dry food	6.09±0.65	7.69±0.61	6.89±0.44		
Mean	5.36±0.44	6.34±0.43		0.124	0.002
Uric Acid renal excretion (mg/kgBW/d)					
Wet food	1.45±0.18	1.81±0.19	1.63±0.13		
Dry food	0.88±0.19	1.37±0.18	1.12±0.13		
Mean	1.16±0.13	1.59±0.13		0.026	0.009
Urea urine concentration (mg/dL)*					
Wet food	2937.9±226.5^A^^b^	3165.9±226.5^A^^b^	3051.9±160.16		
Dry food	6616.5±240.2^B^^a^	9515.2±226.5^A^^a^	8064.1±165.09		
Mean	4777.2±165.09	6339.07±160.16		< .001	< .001
Urea renal excretion (mg/kgBW/d)					
Wet food	890.5±59.87	1254.5±59.87	1007.5±49.06		
Dry food	929.7±59.87	1386.3±73.96	1158.0±53.65		
Mean	910.1±49.06	1320.4±53.65		< .001	0.140
Ammonia urine concentration (mg/dL)					
Wet food	157.2±8.80	147.0±8.80	152.1±6.23		
Dry food	159.1±8.80	164.5±8.80	161.8±6.23		
Mean	158.1±6.23	155.7±6.23		0.787	0.280
Ammonia renal excretion (mg/kgBW/d)					
Wet food	49.2±3.85	60.0±3.85	54.6±2.72		
Dry food	24.4±3.85	28.2±3.85	26.3±2.72		
Mean	36.8±2.72	44.1±2.72		0.067	< .001
Mg intake (mg/kgBW/d)*					
Wet food	7.48±0.45^A^^b^	8.67±0.45^A^^a^	8.09±0.39		
Dry food	11.08±0.45^A^^a^	7.50±0.45^B^^a^	9.29±0.39		
Mean	9.12±0.39	7.76±0.39		0.001	0.001
Mg urine concentration (mg/dL)*			
Wet food	3.07±0.32^A^^b^	3.62±0.29^A^^b^	3.35±0.22		
Dry food	9.21±0.32^B^^a^	11.86±0.32^A^^a^	10.53±0.23		
Mean	6.14±0.23	7.75±0.22		0.001	< .0001
Mg renal excretion (mg/kgBW/d)					
Wet food	1.05±0.12	1.46±0.12	1.26±0.08		
Dry food	1.41±0.12	1.87±0.12	1.64±0.08		
Mean	1.23±0.08	1.66±0.08		0.001	0.004
Cl intake (mg/kgBW/d)*					
Wet food	120.45±4.11^B^^b^	143.46±4.11^A^^b^	131.95±2.90		
Dry food	190.77±4.66^A^^a^	168.97±4.66^B^^a^	180.84±3.30		
Mean	155.61±3.10	157.18±3.10		0.72	< .001
Cl urine concentration (mg/dL)					
Wet food	580.77±81.48	449.96±81.48	515.37±64.55		
Dry food	1272.16±81.48	1197.04±81.48	1234.60±64.55		
Mean	926.47±64.55	823.50±64.55		0.153	< .001
Cl renal excretion (mg/kgBW/d)*					
Wet food	143.74±11.07^B^^b^	187.47±11.07^A^^a^	165.60±9.08		
Dry food	185.34±10.93^A^^a^	187.62±11.61^A^^a^	186.48±9.44		
Mean	164.54±9.12	187.54±9.40		0.015	0.026
K intake (mg/kgBW/d)					
Wet food	52.7±3.77	50.1±3.77	51.4±3.27		
Dry food	86.2±3.77	82.0±3.77	84.1±3.27		
Mean	69.7±3.27	66.1±3.27		0.214	< .001
K urine concentration (mg/dL)					
Wet food	187.4±15.97	151.7±15.97	169.6±11.64		
Dry food	530.5±16.94	532.8±15.97	531.6±11.29		
Mean	358.9±11.64	342.3±11.29		0.313	< .001
K renal excretion (mg/kgBW/d)					
Wet food	57.8±3.03	60.6±3.37	59.2±2.31		
Dry food	75.5±3.03	81.2±3.03	78.3±2.42		
Mean	66.6±2.31	70.9±2.42		0.149	< .001
P intake (mg/kgBW/d)*					
Wet food	94.6±5.49^A^^b^	108.0±5.49^A^^a^	101.19±4.67		
Dry food	141.7±5.49^A^^a^	60.4±5.49^B^^b^	101.07±4.67		
Mean	118.0±4.67	84.2±4.67		< .001	0.975
P urine concentration (mg/dL)*					
Wet food	164.8±12.87^A^^b^	169.0±10.51^A^^a^	166.9±8.31		
Dry food	260.3±10.51^A^^a^	183.5±10.51^B^^a^	221.9±8.31		
Mean	212.5±8.03	176.3±7.43		0.003	< .001
P renal excretion (mg/kgBW/d)*					
Wet food	50.5±2.35^A^^a^	65.1±2.19^A^^a^	57.8±1.60		
Dry food	39.0±2.07^Ab^	29.3±2.19^B^^b^	34.2±1.51		
Mean	44.8±1.56	47.2±1.55		0.848	< .001

^1^ SEM—standard error of the mean (n = 9 cats per food).

^2^ S:P—effect of the starch to protein ratio of the diet.

* Starch to protein ratio * moisture content interaction (P<0.05).

^A,B^ Means in a row without a uppercase letter in common are different (P<0.05).

^a,b^ Means in a column without a lowercase letter in common are different (P<0.05). Comparison valid only within each parameter.

An interaction between food type and starch to protein ratio was observed for urea urine concentration (P<0.05), which was higher for cats fed the high protein dry food, intermediate for animals fed the high starch dry food, and lower for animals fed both wet food formulations (P<0.05). Urea renal excretion, however, only differed between the formulation types and was higher for cats fed the high protein diets than for those fed the high starch diets (P<0.01). The ammonia urine concentration was similar among treatments, and its renal excretion was higher for cats fed the wet foods (P<0.01).

An interaction was observed for Mg intake, with higher values for cats fed the high starch than the high protein dry food, and for high for cats fed with the high protein than the high starch wet food (P<0.05). Mg urine concentration and renal excretion was higher for cats fed dry foods (P<0.05), but an interaction was observed for urinary Mg concentration, which was similar in cats fed the two wet food formulations but higher in cats fed the high-protein dry diet compared to the high-starch dry diet (P<0.05). An interaction was observed for Cl intake, which was higher for cats fed the dry diets than wet formulations, and according to starch to protein ratio higher for high protein than high starch wet food, but higher for high starch than the high protein dry food (P<0.05). The Cl urine concentration was similar among cats fed high protein or high starch formulations, but higher for animals fed the dry foods (P<0.05). Cl renal excretion was similar across high protein formulations, regardless of processing type, but was lower in cats fed the high starch wet diet compared to those fed the high starch dry diet (P<0.05). The intake, urine concentration, and renal excretion of K was similar for both starch to protein ratios, but lower for wet than dry food intake (P<0.05). Similar intake of P was observed for cats fed wet foods, regardless of starch to protein ratio. However, P intake was lower in cats fed the high protein dry food compared to the high protein wet food and lower in cats fed the high starch wet food compared to the high starch dry food (P<0.05). An interaction was observed for urine P concentration (P<0.05), which was higher in cats fed the high starch dry food than in those fed the high starch wet formulation (P<0.05), was similar among high protein formulations, regardless of processing type, and, within dry foods, was higher in the high starch formulation than in the high protein formulation (P<0.05). Phosphorus renal excretion was lower in cats fed dry foods, and within the dry diets, it was higher for the high starch formulation compared to the high protein formulation (P<0.05).

The results of urine RSS for calcium oxalate differed between the Equil and Supersat software estimations (Table 6). Using Equil, the effects of both the diet type and starch content were verified, and the cats fed dry foods and the high-starch formulations presented a higher RSS for calcium oxalate (P<0.01). Utilizing Supersat, no effect of the starch to protein ratio was observed; there was only a diet type effect with a higher RSS for calcium oxalate in cats fed the dry foods (P<0.01). An interaction between food type and starch to protein ratio was observed for urine RSS for struvite (P<0.05), which was higher for cats fed the high protein dry food, intermediate for high starch dry food, and lower for cats fed both wet food formulations (P<0.05). The oxalate urine concentration showed only an effect of the starch to protein ratio; it was higher for cats fed the high starch foods (P<0.01). Oxalate renal excretion, however, was higher for cats fed wet foods than for cats fed dry foods (P<0.01), regardless of the starch to protein ratio. Citrate in urine was higher for cats fed the dry foods (P<0.05), and its renal excretion was higher for animals fed the wet diets and the high protein formulations (P<0.01).

**Table 6:** Urine Relative Supersaturation (RSS) for calcium oxalate and struvite, and oxalate and citrate urine concentration and renal excretion of cats fed experimental diets with different starch to protein ratios and moisture contents.

Item	Starch to protein ratio		Mean		P value	
	High starch	High protein			S:P^2^	Moisture
RSS for calcium oxalate (Calculated with Equil-93)					
Wet food	0.33±0.14	0.10±0.14	0.22±0.12			
Dry food	0.72±0.14	0.39±0.14	0.55±0.12		
Mean	0.53±0.12	0.25±0.12			0.005	0.001
RSS for calcium oxalate (Calculated with Supersat)					
Wet food	0.03±0.11	0.04±0.11	0.03±0.11			
Dry food	0.33±0.11	0.32±0.11	0.32±0.11			
Mean	0.18±0.11	0.18±0.11			0.945	0.001
RSS for struvite (Calculated with Supersat) *					
Wet food	0.02±0.01^A^^b^	0.01±0.01^A^^b^	0.02±0.01			
Dry food	0.06±0.^01^^B^^a^	0.11±0.^01^^A^^a^	0.09±0.01			
Mean	0.04±0.01	0.06±0.01			0.060	< .0001
Oxalate urine concentration (mg/dL)					
Wet food	2.24±0.31	1.50±0.31	1.88±0.27			
Dry food	2.09±0.31	1.18±0.31	1.63±0.27			
Mean	2.17±0.27	1.34±0.27			<0.001	0.234
Oxalate renal excretion (mg/kgBW/d)					
Wet food	0.70±0.09	0.69±0.09	0.69±0.08			
Dry food	0.21±0.09	0.11±0.09	0.16±0.08			
Mean	0.46±0.08	0.40±0.08			0.333	< .001
Citrate urine concentration (mg/dL)					
Wet food	21.5±0.52	22.1±0.52	21.8±0.37			
Dry food	23.3±0.56	24.8±0.56	24.1±0.38			
Mean	22.4±0.38	23.4±0.37			0.063	< .001
Citrate renal excretion (mg/kgBW/d)					
Wet food	7.00±0.33	9.09±0.35	8.04±0.22			
Dry food	3.19±0.29	4.10±0.35	3.64±0.23			
Mean	5.09±0.22	6.60±0.23			<0.001	< .0001

^1^ SEM—standard error of the mean (n = 9 cats per food).

^2^ S:P—effect of the starch to protein ratio of the diet.

* Starch to protein ratio * moisture content interaction (P<0.05).

^A,B^ Means in a row without a uppercase letter in common are different (P<0.05).

^a,b^ Means in a column without a lowercase letter in common are different (P<0.05). Comparison valid only within each parameter.

## Discussion

The results obtained partially confirmed the proposed hypothesis of the study. Cats fed wet foods presented lower RSS for calcium oxalate and struvite than animals fed dry foods, but starch intake influenced oxalate urine concentration and the RSS for calcium oxalate in both diet types, with lower values for high protein formulations. Interestingly, the opposite was verified for struvite RSS, which was lower for cats fed dry foods high in starch than for those high in protein, reinforcing that the prevention of these two types of uroliths requires different dietary approaches [37,38]. It is important to note that only healthy cats that had never presented urinary problems were included in the present study, and it is possible that stone-forming animals might present different responses to the dietary interventions tested here [8,12]. Additionally, it is necessary to consider that the urine of all cats was in the undersaturated zone of both investigated uroliths [12,16], so all four diets evaluated can be considered adequate and in fact was in the range of therapeutic diets for cats with urolithiasis formation [39].

The differences in dry matter intake between diet types could be related to the physical form and moisture content of the wet diets or may only reflect the higher crude fat and energy content of the wet food formulations [40]. Despite the short length of the study, food intake was sufficient for the cats to maintain a constant body weight. The differences in starch, protein and water intake reflected diet composition and the study objectives. Starch intake was significantly higher, as expected, for the high starch diets, approximately 2.6 times greater. However, due to variations in dry matter intake, cats fed the high starch wet food consumed less starch than those fed the high starch dry food. In the high protein formulations, the difference in starch intake was even more pronounced, as both the dry matter intake and starch content were lower in the wet diet. While these variations should be considered when interpreting the data, the differences in starch intake between diet formulations were sufficient to effectively test the study hypothesis.

The total tract apparent digestibility of diets can be considered adequate, particularly for crude protein, suggesting good raw material selection and processing [41,42]. The lower fat, protein and energy digestibility for the high protein in comparison with the high starch wet food may be related to differences in raw material sources. The lower crude protein apparent digestibility might suggest that the tendency for higher ammonia renal excretion in cats fed the high protein wet food is likely related to protein fermentation and ammonia absorption in the colon, which was subsequently excreted by the kidneys. Other differences between diets to consider on data interpretation includes the different raw material matrix and processing types. This was consequent to the use of traditional raw materials and may have the advantage to reflect more closely practical conditions related to cat feeding but have the disadvantage to include potential sources of variation related to the different raw materials on results. However, as the digestibility and water balance are described, the reader can interpret and evaluate the diets adequately.

A similar urine pH of cats fed the experimental diets was achieved by formulating the food BE of each treatment. This is important in studies that propose evaluating urine RSS, as urine pH has a strong influence on results [9,43,44]. Although it is sometimes suggested that high protein diets induce low urine pH due to the acid load arising from sulfur amino acid metabolism [26,45], the present results clearly show that food BE derived from macroelement composition is the predominant factor in acid base status [10,46]. The authors decided to test the study hypothesis on formulations which represent practical diets for cats, and not using semi-purified ingredients that would allow for more close composition of macroelements. Due these, inevitable differences on ash and macroelements were observed between diets, what need to be considered in results interpretation. According to each raw material characteristic, minerals bioavailability might also differ, specially considering the differences in Ca and P bioavailability when derived from bone material or purified sources. These are potential confounding factors of the experimental diets design adopted which need to be considered as limitations on data interpretation.

The lower urine density in cats fed the wet foods in comparison with those fed dry diets was expected, as it has already been described in experiments that compared the influence of dry and wet foods on urine characteristics [8,15,16,27]. The desert origin of cats favors the selection of animals with a high capacity to concentrate urine [47]. Therefore, when fed low moisture dry diets, cats drink little water and compensate by the production of concentrated urine. In the present study, total water intake and urine output of cats fed wet foods were more than double those of cats fed dry foods, highlighting the importance of food moisture content on the water balance of cats [16,48].

Although higher protein intake was associated with greater urine production in cats [5,21,22], this was not observed for dry diets in the current study. For cats fed wet diets, however, higher urine excretion and total water intake were observed for animals fed the high protein formulation. This is explained by the higher urea renal excretion observed for cats fed the high protein formulations, increasing the tonicity and water retention in the glomerular filtrate, resulting in a higher urine volume [21].

Among the components of calcium oxalate, although calcium intake was higher for cats fed the high starch formulations, calcium renal excretion and urine concentration were higher for cats fed the high protein formulas. This is especially evident comparing the two dry formulations, as the high starch dry diet presented elevated Ca in comparison with the high protein dry formulation. As explained previously this differences in Ca content happened because of the used of traditional raw materials and due to the necessity to proper balance food BE, in order to cats had similar urine pH. So, if Ca or Mg be corrected with calcium carbonate of magnesium oxide in the high protein dry food, for example, differences in urine pH, that would be more alkaline in the formula supplemented with these mineral sources [10] would interfere on the results. Due these, the authors prioritized the urine pH, allowing variations on macroelements that need to be proper considered as confounding factors in data interpretation.

Although not planned by authors, this difference in Ca intake allowed an interesting observation since it was possible to see that Ca intake alone is not a predictor of it urinary concentration, and other factors such as Ca bioavailability in the diet or metabolic conditions of the cats related to bone metabolism, acid-base equilibrium, protein intake, or others factors may have important effect on Ca renal excretion [7,26,24,44], that were higher in the present study for cats fed the diets with lower amounts of this macroelement. It is possible, however, that if Ca intake had been similar between the different protein levels, a difference even higher in Ca renal excretion might had been observed. This limits the interpretation of the results of the present study and is a concern that should be considered.

This indicates a true effect of protein increasing calcium renal excretion [49], that happened even with the lower intake of this macroelement. High urine calcium is a risk factor for calcium oxalate urolithiasis in dogs, humans, and cats [7,44], and the results of several studies in humans suggest that high protein intake increases urinary calcium [50]. This effect may be explained by the acidifying effect of some proteins, which results in calcium mobilization from bones due to its buffering effect on intermediary metabolism [51] or may be due to increased intestinal calcium absorption induced by high protein intake [50,52]. In felines, however, Pablack *et al* [24] showed an increased renal excretion and urine concentration of calcium with an increase in dietary protein that was independent of changes in acid-base status, as the urine pH of the cats was similar between treatments (as observed in the present study). This effect was also independent of calcium intestinal absorption, which also did not change in their study [24]. Therefore, a direct effect of protein intake, increasing the glomerular filtration rate and calcium renal excretion might explain the protein effect on urinary calcium in cats, but this would need more studies to be completely characterized. Other authors, however, did not detect differences in calcium renal excretion in cats fed high protein diets [5,7,53] reinforcing the necessity to better understand this relationship.

Regarding food moisture content, cats fed dry diets presented higher calcium renal excretion and a calcium concentration in the urine 3.3 times greater than cats fed the wet foods, which was mainly explained by the differences in urine volume. This was probably an important contributing factor for the high urine RSS for calcium oxalate in cats fed dry diets in the present study [16,48]. For oxalate urine concentration, in contrast, no effect of diet moisture content was observed, which was not an expected result. A previous study compared urine calcium, oxalate and RSS for calcium oxalate in client-owned cats presenting with calcium oxalate urolithiasis. The animals were previously fed a variety of commercial dry diets, and when switched to a canned diet formulated to prevent calcium oxalate uroliths, animals showed a reduction in urinary calcium and RSS for calcium oxalate, but the oxalate urine concentration did not decrease, remaining similar to that observed when the cats were fed the dry diets [8]. This response was the same as that observed in the present study, as the wet diets reduced calcium and RSS of calcium oxalate but not the oxalate urine concentration. This relationship among urine volume and urine oxalate concentration deserves future investigations, attempting to improve the nutritional strategies to prevent this urolith formation.

A strong effect of the starch to protein ratio, however, was observed for the oxalate urine concentration. Previous studies have already shown this effect in high moisture [27] and dry diets for cats [5]. This was probably an important contributing factor for the higher urine RSS for calcium oxalate in cats fed high starch diets, which was more than double that observed for animals fed the high protein formulations in the present study (Equil software). The hypothesis of higher endogenous production of oxalate in cats fed high starch diets, however, was not confirmed, as oxalate renal excretion did not differ between the different starch to protein ratios. For people and mice, sugars are important precursors for oxalate endogenous synthesis [54,55]. Because in cats the enzyme alanine glyoxylate aminotransferase is only localized on mitochondria [56], it is speculated that high starch intake with high intestinal glucose absorption might result in increased oxalate synthesis by the cytoplasmatic enzymes glyoxylate reductase/hydroxypyruvate reductase and L-lactate desidrogenase and the enzyme glycolate dehydrogenase in the peroxisomes [26]. Other published papers on cats, however, failed to confirm this hypothesis, and although they reported higher oxalate urine concentrations, they did not observe increased oxalate renal excretion in cats fed high starch diets [5,27]. A recent study demonstrated that genetic factors, specific variants of in the genes associated with oxalate metabolism, may increase the risk of calcium oxalate formation. Analyzing the serum metabolomic profiles and the genotypes of 445 cats, genetic variants of the enzymes alanine-glyoxylate aminotransferase 2 and 2-oxoarginine was identified, and cats with the genotype AA presented a higher incidence of calcium oxalate compared to the AG and GG genotypes [57]. This increases the complexity of the evaluation of starch intake implication to oxalate renal excretion, and a limitation of the present study is that it is unknown the genotype variation on the cats used in this study.

Oxalate intake and intestinal absorption are not considered important risk factors for increased urine oxalate in cats [58]. Most ingredients used in cat food formulations do not have noticeable amounts of oxalate, and in the presence of calcium, the majority of dietary oxalate is excreted in feces [59]. Therefore, one possible explanation for the lower oxalate in urine is a dilutional effect of high protein in comparison with high starch formulations, which was observed for both the dry and wet foods in the present study.

It was interesting to observe that Equil software indicated effects of both moisture content and starch-to-protein ratio in the urine RSS for calcium oxalate, but Supersat indicated only an effect of moisture content, and no effect of starch or protein intake was verified. When comparing dog and cat urine, Equil and Supersat software was considered equivalent in calculating RSS for calcium oxalate [9]. Therefore, it is difficult to speculate why this difference occurred, particularly given that oxalate was higher in the urine of cats fed the high starch diet, but calcium was higher in the urine of cats fed the high protein diet. If one considers the results of Equil, it is possible to see that the increased urine oxalate was the mandatory effect, as even with the higher Ca in urine, cats fed the high protein formulas had a lower RSS for calcium oxalate.

It is important to note that in the present study the EQUIL-93 version of the Equil software was utilized, a version that was used in several publications on dogs and cats [5,60–62]. However, there is a new version of this software adapted for dogs and cats (EQUIL-HL21) [63], and this should be considered when comparing results between different publications, especially between studies that used different software versions. During the new software version validation, however, although different values was found, authors had shown that both versions present a high correlation each other [63]. Due this, although the use of the old version need to be considered, its utilization is still valid to understand the study hypothesis, i.e. the food moisture and starch intake implications on the cat’s urine RSS.

The urine RSS for struvite was very low in all evaluated cats, and this needs to be considered to interpret the results. These low values are explained by the cat urine pH being generally lower than 6.2 for all diets, when most urinary phosphorus is in the form of monobasic and dibasic phosphate and the force of aggregation is low [23,64]. In fact, this range of urine pH is associated with the undersaturated zone and struvite dissolution in the urinary bladder [9,44,60]. Only for cats fed dry diets was the effect of high protein verified, with higher urine RSS for struvite in comparison with cats fed the high starch formulation. This higher value does not coincide with higher ammonia, which was present in similar amounts in the urine of the cats between diets, and nor be explained by phosphorus concentrations, which in fact were higher in the urine of cats fed the high starch dry food. This difference might be attributed, so to the higher urine pH (although not statistically significant) and the higher magnesium concentration in urine of cats fed the dry diet high protein formulation in comparison to high starch dry food. These results reinforce the potential effect of Mg urine concentration increasing RSS for struvite, probably more important than the protein intake effect on urinary ammonia.

Other macroelements that also accounts for the RSS calculation are Na, K, and Cl. As in the present study practical formulations was used, unfortunately it was not possible to control and balance them to assure similar intakes and urine concentration between cats fed the different diets. This is a limitation that need to be taken into account during data interpretation. However, their effects are mainly related to urine volume and pH [64,65], and at least urine pH was adequately controlled in the present study to mitigate their impact on outcomes.

Due to experimental diets design, inevitable influences of all seven macroelements might be present in the study results. To create less interference authors only equilibrated cations and anions targeting urine pH, as already explained, but opted to do not equilibrate all macroelements to them present similar amounts on diets. The difficult in doing this using practical raw materials includes the necessity of use several different purified sources of cations and anions. Potential implications and difficulties in this approach include challenges in obtaining similar urine pH values, and variation in body macroelement balance and in their renal excretion, especially due to potential differences in their bioavailability and intestinal absorption, that might be higher for the purified mineral sources than for the macroelements of the food matrix [66,67]. This might create other potential interferences that might also reduce results reliability. This is, anyway, important to be considered in data interpretation.

Based on the results, it is possible to conclude that an elevated protein to starch ratio, although it induced higher calcium renal excretion and concentration in urine, reduced urine oxalate concentration and RSS for calcium oxalate in both dry and wet diets. This effect was not attributed to a reduced oxalate endogenous synthesis promoted by the limitation in starch intake, as it did not vary between formulation types, but it was attributed to a dilutional effect in urine promoted by the increased protein intake. However, given the limitations of the present study, further investigations into the effects of protein and starch ratios on the urinary characteristics of cats are recommended. Wet diet intake, although it did not reduce the oxalate concentration in urine, resulted in lower urinary calcium, reducing the urine RSS for calcium oxalate and struvite. As all four diets were inside the undersaturated zone for calcium oxalate and struvite RSS [39], the changes induced by protein to starch ratio and moisture content evaluated was not extensive, at least in health and non-stone-forming cats.

## Supporting information

S1 Table Full data set. (XLSX)
